# The *Cacna1h* mutation in the GAERS model of absence epilepsy enhances T-type Ca^2+^ currents by altering calnexin-dependent trafficking of Ca_v_3.2 channels

**DOI:** 10.1038/s41598-017-11591-5

**Published:** 2017-09-14

**Authors:** Juliane Proft, Yuriy Rzhepetskyy, Joanna Lazniewska, Fang-Xiong Zhang, Stuart M. Cain, Terrance P. Snutch, Gerald W. Zamponi, Norbert Weiss

**Affiliations:** 1 Institute of Organic Chemistry and Biochemistry, Academy of Sciences of the Czech Republic, v.v.i., Prague, Czech Republic; 2 Department of Physiology and Pharmacology, Hotchkiss Brain Institute and Alberta Children’s Hospital Research Institute, Cumming School of Medicine, University of Calgary, Calgary, T2N 4N1 Canada; 3 Michael Smith Laboratories and the Djavad Mowafaghian Centre for Brain Health, University of British Columbia, Vancouver, BC V6T 1Z4 Canada

## Abstract

Low-voltage-activated T-type calcium channels are essential contributors to the functioning of thalamocortical neurons by supporting burst-firing mode of action potentials. Enhanced T-type calcium conductance has been reported in the Genetic Absence Epilepsy Rat from Strasbourg (GAERS) and proposed to be causally related to the overall development of absence seizure activity. Here, we show that calnexin, an endoplasmic reticulum integral membrane protein, interacts with the III-IV linker region of the Ca_v_3.2 channel to modulate the sorting of the channel to the cell surface. We demonstrate that the GAERS missense mutation located in the Ca_v_3.2 III-IV linker alters the Ca_v_3.2/calnexin interaction, resulting in an increased surface expression of the channel and a concomitant elevation in calcium influx. Our study reveals a novel mechanism that controls the expression of T-type channels, and provides a molecular explanation for the enhancement of T-type calcium conductance in GAERS.

## Introduction

Generalized non-motor epilepsies are often associated with an hereditary component^1^. They are characterized by the occurrence of spontaneous convulsive or nonconvulsive seizures with sudden bilateral synchronous spike and wave discharges (SWDs) on the electroencephalogram (EEG)^2^. Absence seizures are a type of nonconvulsive generalized seizures, which involve brief and sudden lapses of consciousness, usually associated with generalized 3–4 Hz SWDs^3^. The Genetic Absence Epilepsy Rat from Strasbourg (GAERS) is a well-validated rodent model of absence epilepsy^4^. Absence-like seizures in GAERS are manifested by spontaneous behavioral arrest and staring, clonic twitching of the vibrissae, and high-amplitude SWDs^5^. As for human generalized non-motor epilepsies, SWDs in GAERS are inherited, and notably segregate with a missense mutation in the gene *Cacna1h* encoding for the voltage-gated Ca_v_3.2 T-type calcium channel^6^.

T-type channels are low-voltage-gated calcium channels that operate near the resting electrical membrane potential of nerve cells^7^. Although a preceding period of hyperpolarization may be required to recruit T-type channels from inactivation, they are typically triggered by subthreshold membrane depolarizations to generate a Ca^2+^ transient which in turn gives rise to high frequency bursts of action potentials that support various forms of neuronal rhythmogenesis^8–11^. These aspects of T-type channel function are of direct relevance to the functioning of the thalamocortical network, a brain circuit that is critically involved in the development and propagation of SWDs^12–14^.

Several lines of evidence support a causal implication of T-type channels in the pathogenesis of epilepsy. First, gain-of-function polymorphisms in the human *CACNA1H* gene segregate in patients with childhood and juvenile absence epilepsy^15–20^. Second, direct inhibition of T-type channels (Ca_v_3.1, Ca_v_3.2 and Ca_v_3.3 isoforms) by small organic molecules reduces thalamic burst firing and suppresses seizures in rodent models of absence, temporal lobe epilepsies^21, 22^, and other types of seizures^23^. Third, T-type calcium channel inhibitors are effective in the treatment of absence seizures in humans^24^. Finally, T-type Ca^2+^ conductances are elevated in thalamic neurons of several rodent models of absence epilepsy^25–27^, whereas mice lacking the Ca_v_3.1 T-type channel exhibit increased resistance to absence seizures^28^. The causal link between a primary elevation of T-type Ca^2+^ conductances and the development of absence epilepsy is further supported by the observation that genetic enhancement of Ca_v_3.1 channel expression in mice is sufficient to induce an epileptic phenotype^29^.

The GAERS *Cacna1h* missense mutation results in a splice-variant specific gain-of-function of Ca_v_3.2 currents that exhibit significantly faster recovery from channel inactivation and greater charge transference during high-frequency bursts^6^. However, consistent with the polygenic nature of generalized non-motor epilepsies the *Cacna1h* missense mutation does not by itself entirely account for absence seizure activity in GAERS. Other reported alterations in GAERS include elevated levels of thalamic Ca_v_3.2 mRNA expression^30^ and whole cell T-type currents^25^, although the genetic and molecular mechanisms by which upregulation of T-type channel activity might occur in GAERS and other rodent models of absence epilepsy remain unknown.

It is well described that calnexin, a type I endoplasmic reticulum integral membrane protein and molecular chaperone, is responsible for the folding, quality control and sorting of newly-synthetized (glyco)proteins^31^. Although T-type channels undergo asparagine (N)-linked glycosylation^32–35^, the role of calnexin in the biogenesis and sorting of T-type channels is not understood. Here we show that calnexin binds to and modulates trafficking of Ca_v_3.2 channels to the cell surface by altering the retention of the channel in the endoplasmic reticulum (ER). In addition, we show that calnexin-dependent regulation of Ca_v_3.2 channels is disrupted by the GAERS mutation, thereby leading to an increased surface expression of T-type channels. These results thus reveal a mechanism for the enhanced T-type Ca^2+^ conductance in GAERS, and provide new fundamental knowledge into the biogenesis and molecular trafficking of T-type channels.

## Results

### Calnexin associates with Ca_v_3.2 to modulate channel expression and function

**Figure 1:**
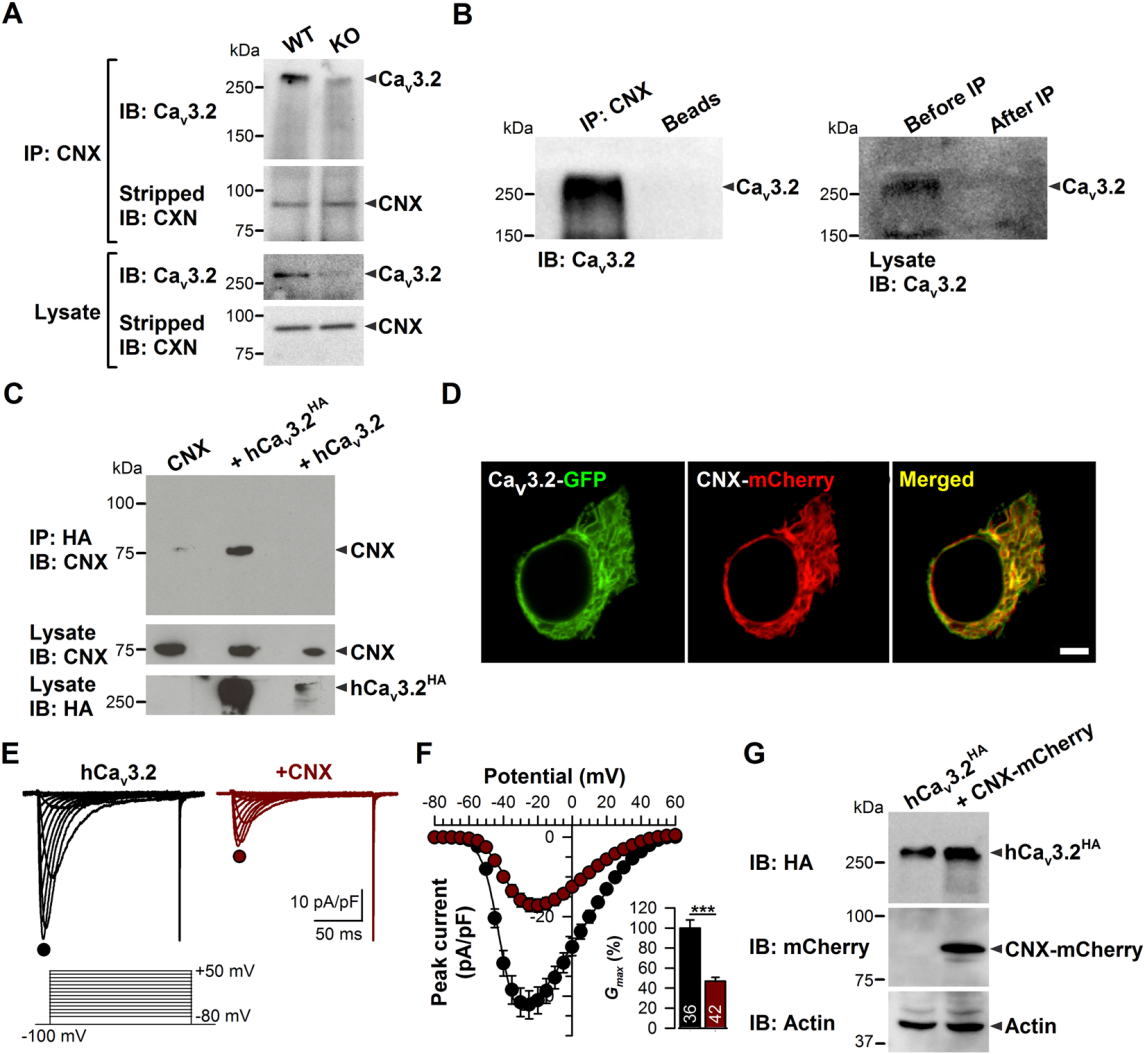
Calnexin associates with Ca_v_3.2 channels and modulates T-type currents. (**A**) Co-immunoprecipitation of Ca_v_3.2 from WT versus Ca_v_3.2 KO mouse brain homogenates with specific anti-calnexin antibody. (**B**) Co-immunoprecipitation of Ca_v_3.2 from rat brain homogenate with specific calnexin antibody. (**C**) Co-immunoprecipitation of calnexin from tsA-201 cells co-transfected with HA-tagged human Ca_v_3.2 channel (hCa_v_3.2-HA). The upper panel shows the result of the co-immunoprecipitation of calnexin with hCa_v_3.2-HA using an anti-HA antibody. The lower panels show the immunoblots of CNX and hCa_v_3.2-HA using an anti-CNX and anti-HA antibody, respectively. (**D**) Confocal images of live tsA-201 cells expressing hCa_v_3.2-GFP channels (green, left column) along with mCherry-tagged CNX (red, middle column). The overlaid image is also shown (right column). (**E**) Representative Ba^2+^ current traces recorded from hCa_v_3.2-HA- (left panel) and hCa_v_3.2-HA/CNX-expressing tsA-201 cells (right panel) in response to 150-ms depolarizing steps varied from −80 to +50 mV from a holding potential of −100 mV. (**F**) Corresponding mean current/voltage relationships for hCa_v_3.2-HA- (filled circles, *n* = 36) and hCa_v_3.2-HA/CNX-expressing tsA-201 cells (open circles, *n* = 42). The *inset* indicates the corresponding maximum normalized slope conductance *G*_max_. (**G**) Immunoblot of hCa_v_3.2-HA from lysates of tsA-201 cells co-transfected with mCherry-tagged CNX (CNX-mCherry). The upper panel shows the result of the immunoblot of hCa_v_3.2-HA using an anti-HA antibody. The middle panel shows the immunoblot of CNX-mCherry using an anti-mCherry antibody. Actin was used as loading control (lower panel). Data are presented as mean ± SEM and were analyzed by Student’s unpaired *t* test; ***p < 0.001.

To determine whether T-type channels and calnexin associate at the protein level, we performed co-immunoprecipitation experiments of Ca_v_3.2 with calnexin from wild type (WT) versus Ca_v_3.2 knock out (KO) brains. As shown in Fig. 1A, a specific anti-calnexin antibody precipitated Ca_v_3.2 from WT mouse brain homogenate, suggesting the existence of T-type channel/calnexin complexes in neuronal tissue. Note that the immunoprecipitated reactive species above 250 KDa that corresponds to the Ca_v_3.2 channel is not present in the co-immunoprecipitation performed from Ca_v_3.2 KO brain, thus demonstrating the specificity of the anti-Ca_v_3.2 antibody used in these experiments. Furthermore, western blot analysis of Ca_v_3.2 from brain homogenate before and after immunoprecipitation indicated that most of Ca_v_3.2 channels are immunoprecipitated by calnexin (Fig. 1B). In contrast, Ca_v_3.2 does not specifically interact with calreticulin and binding immunoglobulin protein (BiP), two other endoplasmic reticulum proteins known to interact with newly synthesized proteins (Fig. S1). The Ca_v_3.2/calnexin interaction was also observed in tsA-201 cells expressing a recombinant HA-tagged human Ca_v_3.2 channel (HA-hCa_v_3.2) (Fig. 1C) where the channel is highly colocalized with calnexin (Fig. 1D). To investigate the impact of Ca_v_3.2/calnexin interaction on channel function, we performed whole-cell patch-clamp recordings of T-type currents. The co-expression of calnexin with Ca_v_3.2 channels in tsA-201 cells revealed a substantial decrease of T-type currents (Fig. 1E). For instance, in response to a depolarizing pulse to −20 mV, the mean peak Ba^2+^ current density was decreased by 58% (*p* < 0.001) in calnexin-overexpressing cells (−17.3 ± 1.7 pA/pF, *n* = 42) as compared to control cells (−41.1 ± 3.4 pA/pF, *n* = 36) (Fig. 1F). The maximum slope conductance (Fig. 1F, inset) was reduced by 53% (p < 0.001) in calnexin-overexpressing cells (331 ± 28 pS/pF, *n* = 42) as compared to control cells (706 ± 55 pS/pF, *n* = 36). Similar results were obtained with hCa_v_3.1 and hCa_v_3.3 channels (Fig. S2). Immunoblot analysis of HA-hCa_v_3.2 revealed that the decreased T-type conductance in calnexin-overexpressing cells was not accompanied by a diminution of the total expression levels of the channel (Fig. 1G). In contrast, nonstationary noise analysis of T-type currents revealed a significant decrease by 76% (p < 0.001) of the number of functional channels expressed at the cell surface (*N*) in calnexin-overexpressing cells (74.8 ± 37.6 channel/pF, *n* = 11) as compared to control cells (311.8 ± 38.6 channel/pF, *n* = 12) (Fig. S3A–C). However, the unitary conductance (*γ*) of hCa_v_3.2 channels was enhanced ~2.5 fold (p < 0.01) in cells overexpressing calnexin (4.0 ± 0.5 pS, *n* = 12) as compared to control cells (1.6 ± 0.2 pS, *n* = 11), without additional effects on the opening probability (*Po*) of the channel (Fig. S3C). It is worth noting that the unitary channel conductance was obtained from the noise analysis of whole cell current traces recorded in the presence of 5 mM Ba^2+^ and is thus lower than the unitary conductance obtained from direct single channel recordings that are usually performed using a high concentration of charge carrier (routinely 110 mM Ca^2+^ or Ba^2+^). The unitary conductance of 1.6 pS in the absence of overexpressed calnexin is consistent with previous studies using similar recording conditions and analysis^36^. In addition, we observed small alterations in some of the biophysical properties of the channel. For instance, activation and inactivation kinetics of Ca_v_3.2 at −20 mV were slightly slower (*p* = 0.0295 and *p* = 0.0066, respectively) in calnexin-expressing cells (τ_activ_ = 5.5 ± 0.6 ms; τ_inac_ 22.5 ± 1.2 ms, *n* = 11) as compared to control cells (τ_activ_ = 4.0 ± 0.3 ms; τ_inac_ = 18.3 ± 0.8 ms, *n* = 12) and we observed a 5 mV depolarizing shift (*p* < 0.0001) of the voltage-dependence of activation in calnexin-expressing cells (−35.2 ± 0.6 mV, *n* = 11) as compared to control cells (−40.0 ± 0.6 mV, *n* = 12).

Altogether, these data support the notion that calnexin participates in the maturation of the hCa_v_3.2 channel and controls its expression at the cell surface.

### Molecular determinants of calnexin interactions with hCa_v_3.2 channels

**Figure 2:**
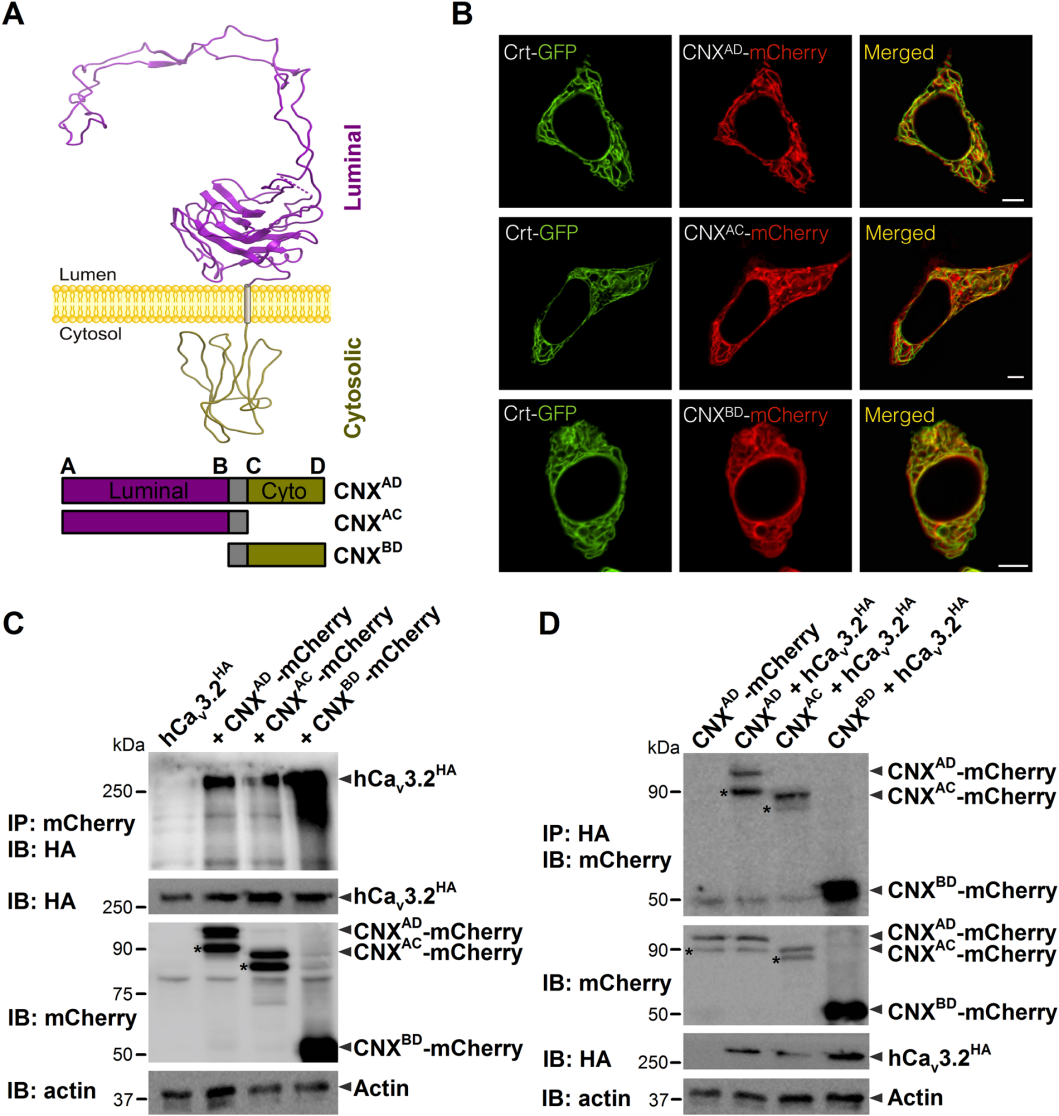
Calnexin associates with hCa_v_3.2 channels via multiple interactions. (**A**) Schematic representation of the topology of CNX (top panel) and the different CNX constructs used including CNX full-length (CNX^AD^), CNX lacking the cytosolic domain (CNX^AC^), and CNX lacking the luminal domain (CNX^BD^) (bottom panel). (**B**) Confocal images of living tsA-201 cells expressing GFP-tagged calreticulin (green, left column) along with mCherry-tagged CNX constructs (red, middle column). Overlaid images are also shown (right column). (**C**) Co-immunoprecipitation of hCa_v_3.2-HA from tsA-201 cells co-transfected with mCherry-tagged CNX deletion constructs. The upper panel shows the result of the co-immunoprecipitation of hCa_v_3.2-HA with CNX-mCherry constructs using an anti-mCherry antibody. Middle panels show the immunoblots of hCa_v_3.2-HA and CNX-mCherry constructs using anti-HA and anti-mCherry antibodies, respectively. Actin was used as loading control (lower panel). (**D**) Co-imunoprecipitation of CNX-mCherry constructs with hCa_v_3.2-HA. The upper panel shows the result of the co-immunoprecipitation of CNX-mCherry constructs with hCa_v_3.2-HA using an anti-HA antibody. Middle panels show the immunoblots of CNX-mCherry constructs and hCa_v_3.2-HA using anti-mCherry and anti-HA antibodies, respectively. Actin was used as loading control (lower panel). *CNX cleaved from the ER signal peptide.

**Figure 3:**
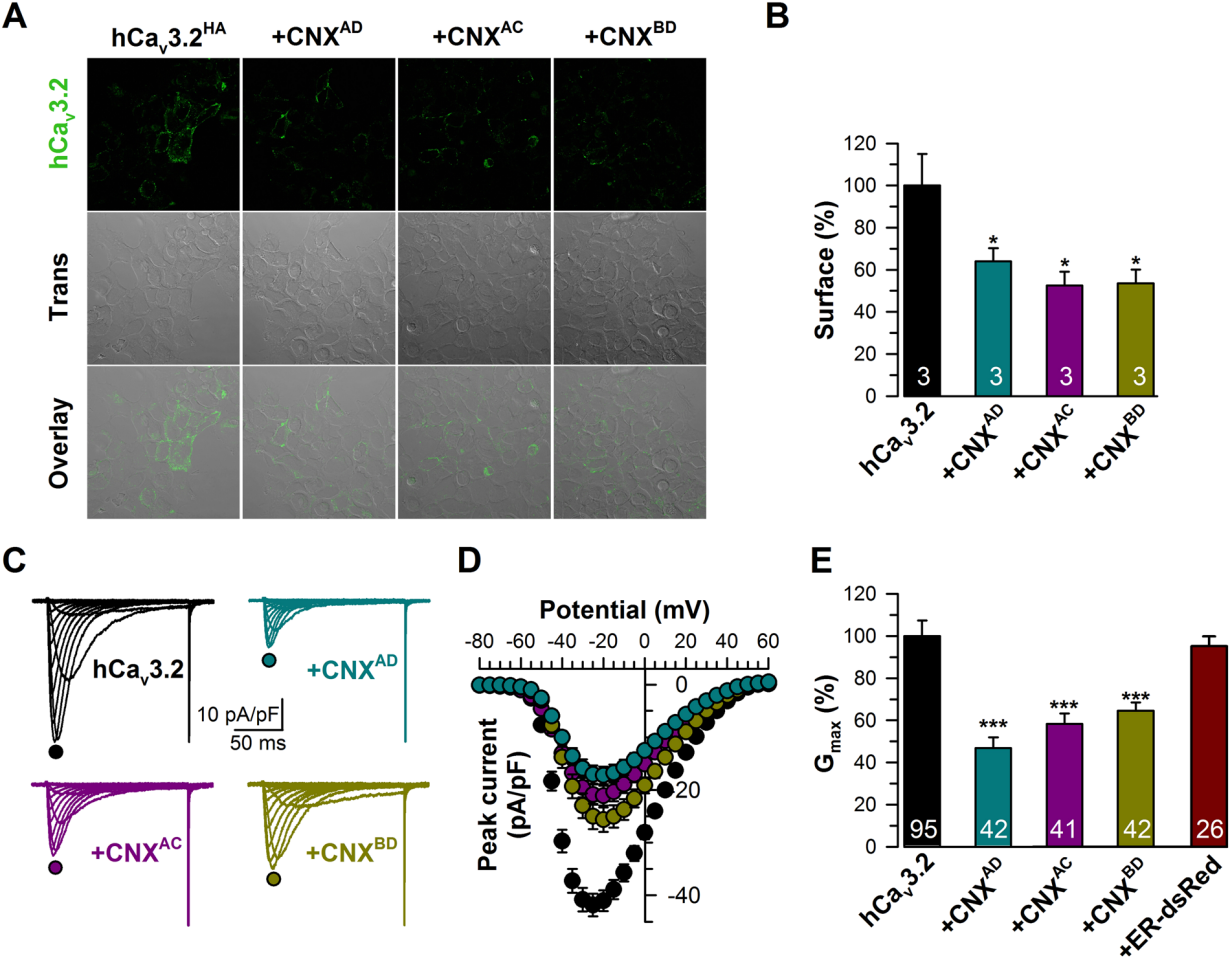
Calnexin contains an ER retention domain that decreases surface expression of hCa_v_3.2 channels. (**A**) Confocal images of non-permeabilized tsA-201 cells expressing hCa_v_3.2-HA along with CNX constructs and stained for hCav3.2-HA (green) using a primary anti-HA antibody. (**B**) Corresponding mean surface expression of hCa_v_3.2-HA. (**C**) Representative Ba^2+^ current traces recorded from tsA-201 cells expressing hCa_v_3.2-HA channels alone (top left panel) or in combination with CNX full-length (CNX^AD^, top right panel), CNX missing the cytosolic domain (CNX^AC^, bottom left panel), or CNX lacking the luminal domain (CNX^BD^, bottom right panel) in response to 150-ms depolarizing steps varied from −80 to +50 mV from a holding potential of −100 mV. (**D**) Corresponding mean current/voltage relationships for hCa_v_3.2-HA expressed alone (black circles, *n* = 95) and in combination with CNX^AD^ (blue circles, *n* = 42), CNX^AC^ (purple circles, *n* = 41), and CNX^BD^ (green circles, *n* = 42). (**E**) Corresponding mean maximum slope conductance *G*_max_ expressed in percentage of hCa_v_3.2-HA-expressing cells. Note that co-expression of the ER-dsRed as no significant effect on hCa_v_3.2-HA currents (dark red bar, *n* = 26). Data are presented as mean ± SEM and were analyzed by Student’s unpaired *t* test; NS not significant, ***p < 0.001.

**Figure 4:**
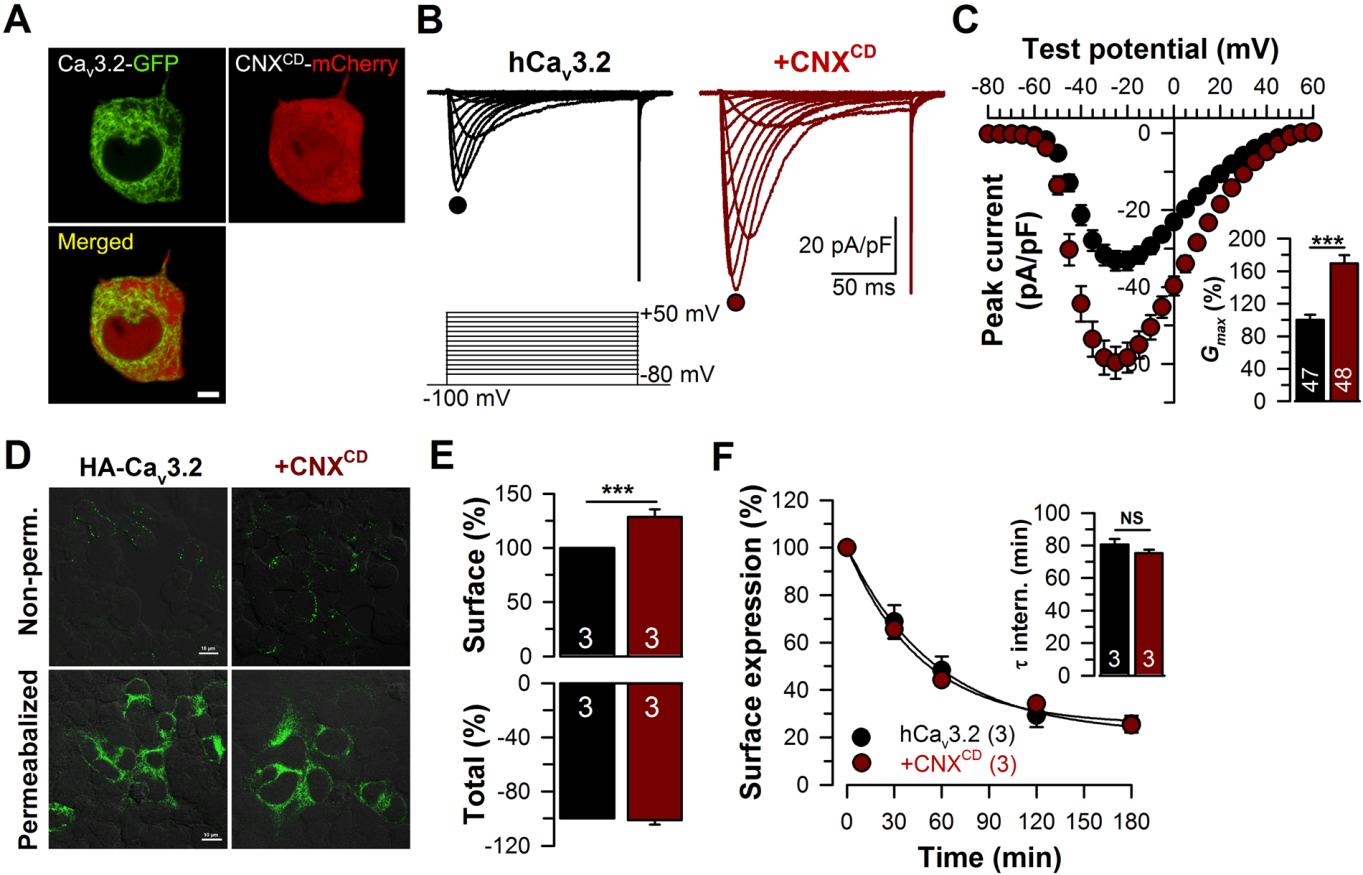
Uncoupling of hCa_v_3.2 from the calnexin C-tail increases surface expression of the channel. (**A**) Confocal images of living tsA-201 cells expressing hCa_v_3.2-GFP channels (green, top left pannel) along with the mCherry-taged cytosolic domain of CNX (CNX^CD^-mCherry) (red, top right panel). Overlaid images are also shown (bottom left panel). Note that in contrast to the CNX^BD^-mCherry construct (Fig. 3B), the CNX^CD^-mCherry construct that does not contain the ER transmembrane domain shows a diffuse expression pattern consistent with a soluble protein. (**B**) Representative Ba^2+^ current traces recorded from hCa_v_3.2-HA- (left panel) and hCa_v_3.2-HA/CNX^CD^-GFP-expressing tsA-201 cells (right panel) in response to 150-ms depolarizing steps varied from −80 to +50 mV from a holding potential of −100 mV. (**C**) Corresponding mean current/voltage relationships for hCa_v_3.2-HA- (black circles, *n* = 47) and hCa_v_3.2-HA/CNX^CD^-GFP-expressing cells (red circles, *n* = 48). The *inset* indicates the corresponding normalized maximum slope conductance *G*_max_. Data are presented as mean ± SEM and were analyzed by Student’s unpaired *t* test; ***p < 0.001. (**D**) Low magnification confocal images of non-permeabilized (top panels) and permeabilized (bottom panels) tsA-201 cells expressing hCa_v_3.2-HA alone (left panels) and together with calnexin C-tail (CNX^CD^) (right panels) and stained for hCa_v_3.2-HA (green) using a primary anti-HA antibody. (**E**) Corresponding mean surface and total expression of hCa_v_3.2-HA channels expressed alone (black, *n* = 3) and in combination with CNX^CD^ (red, *n* = 3), assessed from non-permeabilized and permeabilized cells, respectively. (**F**) Internalization kinetics at 37 °C of hCa_v_3.2-HA channels expressed alone (black, *n* = 3) and in combination with CNX^CD^ (red, *n* = 3). The *inset* indicates the time constant τ of internalization.

To determine the molecular determinants of calnexin/Ca_v_3.2 interaction, we developed mCherry-tagged full-length calnexin (CNX^AD^) and deletion mutants of calnexin corresponding to the luminal (CNX^AC^) and cytosolic regions of the protein (CNX^BD^) (Fig. 2A). The transmembrane domain of calnexin was conserved in all constructs in order to preserve ER localization. Confocal images of live tsA-201 cells expressing GFP-tagged calreticulin together with mCherry-tagged calnexin-deletion mutants confirmed that the fusion proteins are effectively expressed in the ER compartment (Fig. 2B). A similar colocalization of mCherry-tagged CNX constructs was also observed with GFP-tagged BiP and with an ER-targeted GFP construct (data not shown). We then investigated by co-immunoprecipitation which region of calnexin is required for the interaction with hCa_v_3.2 channels. As shown in Fig. 2C, the mCherry-tagged luminal (CNX^AC^) and cytosolic regions of calnexin (CNX^BD^) both immunoprecipitated HA-hCa_v_3.2 from tsA-201 cells. Conversely, HA-tagged hCa_v_3.2 immunoprecipitated mCherry-tagged CNX^AC^ and CNX^BD^ (Fig. 2D). To examine the impact of Ca_v_3.2/calnexin interactions on the trafficking of T-type channels at the plasma membrane, we performed surface immunostaining of HA-hCa_v_3.2 expressed in tsA-201 cells along with calnexin-deletion mutants. Confocal images of surface HA-hCa_v_3.2 are shown in Fig. 3A. Surface expression of HA-hCa_v_3.2 was reduced by 48% (p < 0.05) and 47% (p < 0.05) in cells expressing CNX^AC^ and CNX^BD^, respectively (Fig. 3B). In contrast, surface expression of the membrane-targeted form of GFP (Lck-GFP), which does not interact with calnexin, was unaffected by the co-expression of calnexin, indicating that the effect of calnexin on surface expression of the channel is not mediated by unspecific saturation of the cellular machinery (Fig. S4). Consistent with a decreased surface expression of hCa_v_3.2 channels in the presence of calnexin constructs, T-type currents were significantly reduced (Fig. 3C). For instance, the mean peak Ba^2+^ current recorded in response to a depolarizing pulse to −20 mV was decreased by 49% (*p* < 0.001) and 38% (*p* < 0.001) in cells expressing CNX^AC^ (−21.1 ± 1.9 pA/pF, *n* = 41) and CNX^BD^ (−25.7 ± 2.3 pA/pF, *n* = 42), respectively, as compared to control cells (−41.2 ± 2.0 pA/pF, *n* = 95) (Fig. 3D). The maximum slope conductance was reduced by 43% (*p* < 0.001) and 31% (*p* < 0.001) in cells expressing CNX^AC^ (415 ± 35 pS/pF, *n* = 41) and CNX^BD^ (489 ± 39 pS/pF, *n* = 42), respectively, as compared to control cells (732 ± 34 pS/pF, *n* = 95) (Fig. 3D). In contrast, expression of an ER-targeted construct that does not have biological function (ER-dsRed) had no significant effect on T-type currents, indicating that the decreased conductance observed in the presence of calnexin-deletion mutants is not mediated by a non-specific stress of the ER that could have resulted from overexpression, but rather from the specific binding of calnexin regions with hCa_v_3.2 and the retention of the channel in the ER. This notion is further supported by our observation that uncoupling of hCa_v_3.2 channels from calnexin by co-expression of the mCherry-calnexin C-tail (CNX^CD^) fusion protein produced a robust increase of T-type currents (Fig. 4A,B). For instance, in response to a depolarizing pulse to −20 mV, the mean peak Ba^2+^ current density was increased by 76% (*p* < 0.001) in cells expressing CNX^CD^ (−58.4 ± 3.9 pA/pF, *n* = 48) as compared to control cells (−33.2 ± 2.4 pA/pF, *n* = 47) (Fig. 4C). The maximum slope conductance (Fig. 4C, inset) was increased by 70% (p < 0.001) in CNX^CD^ -expressing cells (1028 ± 61 pS/pF, *n* = 48) as compared to control cells (607 ± 41 pS/pF, *n* = 47). Consistent with the notion that the increased T-type conductance in the presence of the calnexin C-tail arose from the uncoupling of Ca_v_3.2 from calnexin and thus increased trafficking of the channel to the plasma membrane, the surface expression of HA-tagged Ca_v_3.2 channels assessed by immunostaining was increased by 30% (*p* < 0.001) in cells expressing the calnexin C-tail (Fig. 4D-E). In contrast, calnexin C-tail had no effect on the total expression of the channel protein. Because the elevated T-type conductance in the presence of calnexin C-tail could also have resulted from a decrease of channel internalization rather than from an increase of the trafficking of the channel to the plasma membrane^37^, we assessed internalization kinetics of HA-hCa_v_3.2 channels. The time constant of internalization of HA-hCa_v_3.2 channels at 37 °C was unaffected in cells expressing calnexin C-tail (75.3 ± 2.2 min, *n* = 3) as compared to control cells (80.8 ± 3.3 min, *n* = 3) (Fig. 4F).

Collectively, these results indicate that various regions of calnexin are engaged in the interaction with hCa_v_3.2 channels. Of particular interest is the demonstration that interactions with the cytosolic calnexin C-tail are involved in the retention of hCa_v_3.2 in the ER, thus restricting surface expression of the channel.

### The calnexin C-tail associates with the III-IV linker region of hCa_v_3.2 channels

**Figure 5:**
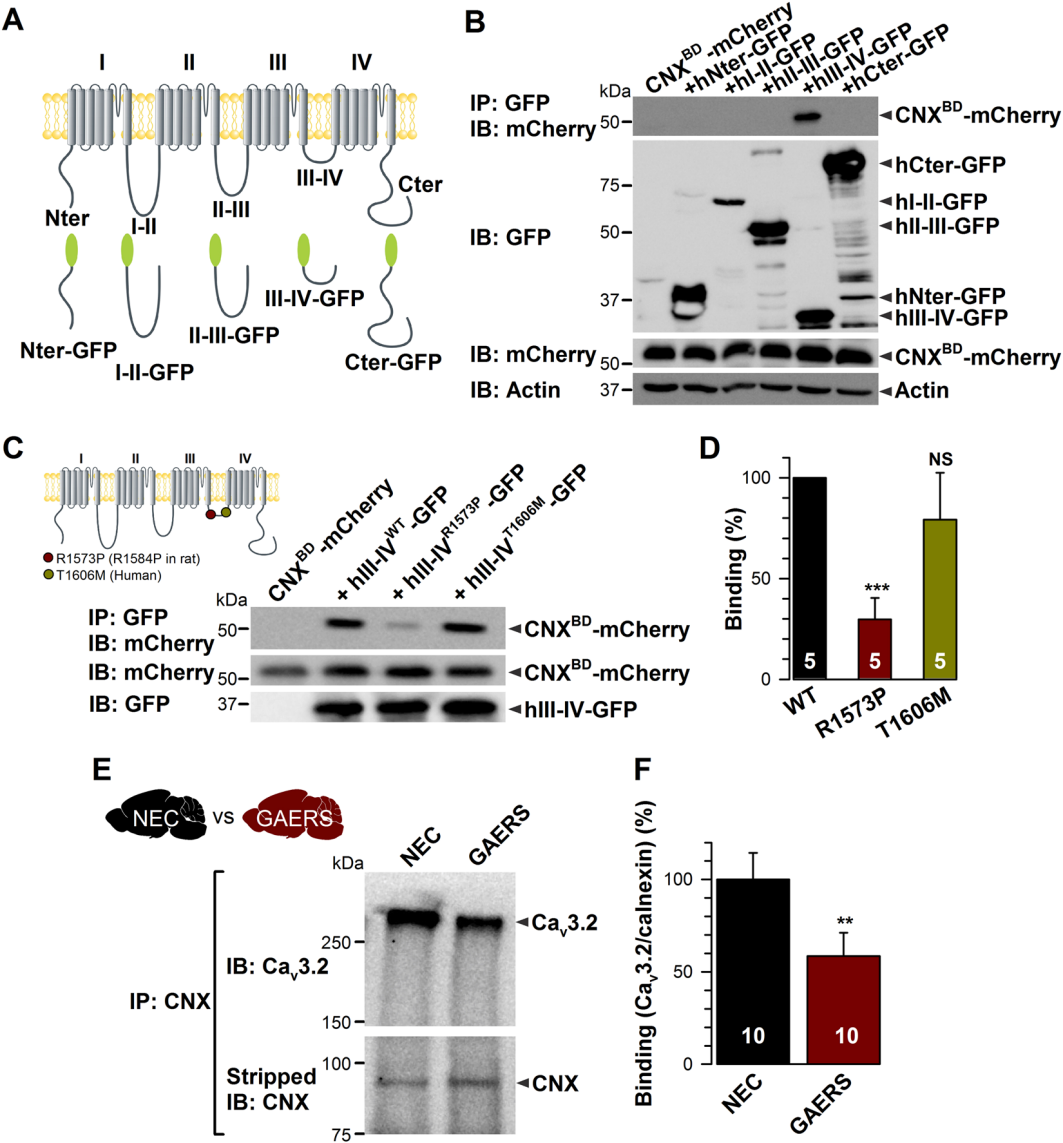
The calnexin C-tail associates with the intracellular III-IV linker of hCa_v_3.2. (**A**) Schematic representation of the different constructs corresponding to the main intracellular regions of hCa_v_3.2. (**B**) Co-immunoprecipitation of CNX C-tail (CNX^BD^-mCherry) from tsA-201 cells with the main intracellular domains of hCa_v_3.2. The upper panel shows the result of the co-immunoprecipitation of CNX^BD^-mCherry with hCa_v_3.2-GFP constructs using an anti-GFP antibody. Middle panels show the immunoblots of hCa_v_3.2-GFP constructs and CNX^BD^-mCherry using anti-GFP and anti-mCherry antibodies, respectively. Actin was used as loading control (lower panel). (**C**) Co-immunoprecipitation of CNX Ctail (CNX^BD^-mCherry) from tsA-201 cells with hCa_v_3.2 III-IV linkers carrying the rat GAERS and human epilepsy mutations. The upper panel shows the result of the co-immunoprecipitation of CNX^BD^-mCherry with hCav3.2 III-IV-GFP variants using an anti-GFP antibody. Lower panels show the immunoblots of CNX^BD^-mCherry and hCa_v_3.2 III-IV-GFP using anti mCherry and anti-GFP antibodies, respectively. *Inset* shows the location of the rat GAERS (red circle) and human epileptic mutations (green circle) in the human Ca_v_3.2 channel. Note that the GAERS R1584P mutation in the rat Ca_v_3.2 channel is equivalent to the R1573P mutation in the human channel. (**D**) Corresponding mean binding values of CNX^BD^-mCherry on hIII-IV^WT^ (black bar) and on hIII-IV^R1573P^ (GAERS, red bar) and hIII-IV^T1606M^ (green bar) variants (*n* = 7). The CNX^BD^-mCherry signal was quantified by densitometry using ImageJ software and normalized to the expression level of the corresponding III-IV-GFP used to immunoprecipitate calnexin. (**E**) Co-immunoprecipitation of Ca_v_3.2 from NEC versus GAERS brain homogenates with specific anti-calnexin antibody. Corresponding normalized mean binding values of Ca_v_3.2 on calnexin in NEC (black bar) and GAERS brains (red bar). The Ca_v_3.2 signal was normalized to the corresponding immunoprecipitated calnexin signal. Data are presented as mean ± SEM and were normalized to total C-tail expression levels and analyzed by one-way ANOVA with Tukey’s post tests; NS not significant, **p < 0.01, ***p < 0.001.

To identify the Ca_v_3.2 molecular determinants of calnexin interactions, we assessed the ability of different intracellular regions of hCa_v_3.2 to interact with calnexin C-tail. The main intracellular regions of hCa_v_3.2 channels fused to the GFP (Fig. 5A) were expressed in tsA-201 cells together with the mCherry-tagged calnexin C-tail (mCherry-CNX^BD^). As shown in Fig. 5B, the calnexin C-tail was specifically immunoprecipitated with the III-IV linker region of hCa_v_3.2. A similar observation was made with the calnexin C-tail deleted of the ER transmembrane domain (mCherry-CNX^CD^) indicating that the transmembrane domain is not required for this interaction (Fig. S5). Interestingly, the GAERS mutation correlating with absence seizures in GAERS is located within exon 24 of *Cacna1h* that encodes the proximal region of the III-IV linker. This missense mutation produces an arginine to proline switch (R1584P) that could potentially affect the electrostatic potential and/or tertiary structure of the III-IV linker and alter protein-protein interactions. To investigate whether the binding of calnexin C-tail is altered by the GAERS mutation, we introduced the homologous mutation (R1573P) into the human III-IV linker. In addition, we generated a III-IV linker construct containing a human mutation (T1606M) previously identified in patients with generalized non-motor epilepsies^2^° (Fig. 5C, inset). Co-immunoprecipitations from tsA-201 cells expressing the GFP-tagged III-IV variants together with mCherry-tagged calnexin C-tail (mCherry-CNX^BD^) revealed a substantially decreased interaction of the calnexin C-tail with the III-IV variant containing the GAERS mutation (Fig. 5C). Indeed, the ability of III-IV^R1573P^ to immunoprecipitate calnexin C-tail was decreased by 70% (p < 0.001) as compared to the wild-type III-IV linker (Fig. 5D). In contrast, the T1606M mutation did not have a significant effect, although there did appear to be a trend towards a weakening interaction. To further support the notion that Ca_v_3.2/calnexin interaction is altered by the GAERS mutation, we performed co-immunoprecipitation experiments of Ca_v_3.2 with calnexin from GAERS brain compared to its non-epileptic control (NEC) strain. Consistent with co-immunoprecipitation studies using calnexin C-tail and Ca_v_3.2 III-IV linker, co-immunoprecipitations from GAERS brain homogenates revealed a substantial decrease of the interaction of Ca_v_3.2 with calnexin (Fig. 5E) in GAERS brain by 42% (p < 0.01) compared to NEC brain (Fig. 5F).

Altogether, these data identified the III-IV linker region of Ca_v_3.2 as the channel region engaged in the interaction with calnexin C-tail, and indicate that this interaction is disrupted by the GAERS Ca_v_3.2 missense mutation.

### The GAERS mutation alters surface expression of Ca_v_3.2 channels by modulating calnexin interactions

**Figure 6:**
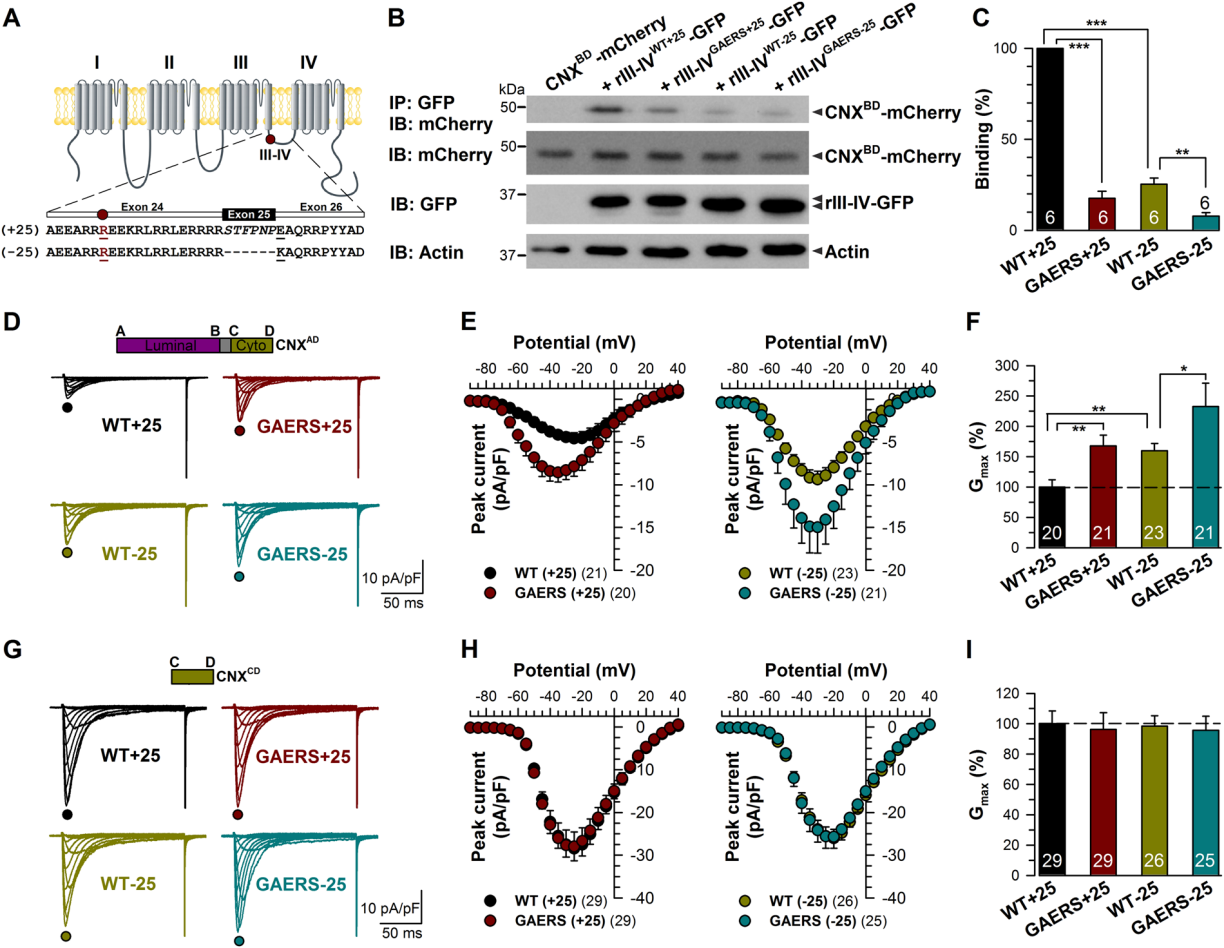
The GAERS epilepsy mutation alters surface trafficking of Ca_v_3.2 channels. (**A**) Schematic representation of the rat Ca_v_3.2 III-IV linker variants. The exon 25 contained in the III-IV linker is alternatively spliced to generate rCa_v_3.2 (+25) and rCa_v_3.2 (−25) channels. The GAERS mutation R1584P located upstream the exon 25 is highlighted in red. (**B**) Co-immunoprecipitation of CNX Ctail (CNX^BD^-mCherry) from tsA-201 cells with rat Ca_v_3.2 III-IV-GFP variants. The upper panel shows the result of the co-immunoprecipitation CNX^BD^-mCherry with rCa_v_3.2 III-IV-GFP variants using an anti-GFP antibody. Middle panels show the immunoblots of CNX^BD^-mCherry and rCa_v_3.2 III-IV-GFP using anti mCherry and anti-GFP antibodies, respectively. Actin was used as loading control (lower panel). (**C**) Corresponding mean binding values of CNX^BD^-mCherry on rCa_v_3.2 WT- (black bar) and GAERS-III-IV linker (red bar) containing exon 25, and on WT- (green bar) and GAERS-III-IV linker (blue bar) lacking of the exon 25. The data were normalized to total C-tail expression levels. (**D** and **E**) Mean current/voltage relationship of T-type currents recorded from tsA-201 cells expressing rCa_v_3.2^WT^(+25) (black circles, *n* = 20), rCa_v_3.2^GAERS^(+25) (red circles, *n* = 21), rCa_v_3.2^WT^(−25) (green circles, *n* = 23), and rCa_v_3.2^GAERS^(−25) (blue circles, *n* = 21). (**F**) Corresponding normalized mean maximum slope conductance *G*_max_ values. (**G**,**H** and **I**) Same as in (**D** and **F**) but for cells expressing the calnexin C-tail peptide. Data are presented as mean ± SEM and were analyzed by one-way ANOVA with Tukey’s posttests; NS not significant, *p < 0.05, **p < 0.01, ***p < 0.001.

It is possible that the decrease in calnexin C-tail binding to the human Ca_v_3.2 III-IV linker region carrying the equivalent GAERS mutation may be channel splice variant specific. We thus examined the effect of the GAERS mutation on the III-IV linker of the two major Ca_v_3.2 channel splice variants expressed in the rat thalamus, Ca_v_3.2 (+25) and Ca_v_3.2 (−25), which differ in the presence or absence of the exon 25. This exon encodes a short stretch of six amino acid residues and is located 14 residues downstream the GAERS mutation (Fig. 6A). Consistent with our previous observation, the binding of calnexin C-tail to the rIII-IV linker (+25) containing the GAERS mutation was significantly decreased by 82% (p < 0.001) compared to the wild-type rIII-IV (+25) (Fig. 6B and C). In addition, the binding of calnexin C-tail to the short wild-type rIII-IV linker variant (−25) was also significantly decreased by 75% (p < 0.001). Introducing the GAERS mutation in the rIII-IV linker (−25) variant produced an additional 69% decrease (p < 0.01) in binding to the calnexin C-tail compared to wild-type rIII-IV (+25).

To establish whether the decrease in the calnexin C-tail interaction with the rCa_v_3.2 III-IV linker also alters surface expression of full-length rCa_v_3.2 channels, we examined T-type currents in cells expressing various channel variants together with overexpressed full-length calnexin. T-type currents recorded from tsA-201 cells expressing the rCa_v_3.2 (+25) channel isoform were significantly increased when the GAERS mutation was introduced (Fig. 6D and E). The maximal conductance was increased by 67% (p < 0.01) in rCa_v_3.2^GAERS^ (+25)-expressing cells (283 ± 30 pS/pF, *n* = 21) as compared to cells expressing the wild-type rCa_v_3.2 (+25) channel (169 ± 20 pS/pF, *n* = 20) (Fig. 6F). In addition, and consistent with our observation that binding of calnexin C-tail is enhanced in the presence of exon 25, T-type currents recorded from cells expressing the rCa_v_3.2 (−25) channel variant were elevated (Fig. 6D and E). For instance, the maximal conductance was increased by 60% (p < 0.01) in rCa_v_3.2 (−25)-expressing cells (270 ± 20 pS/pF, *n* = 23), and by 133% (p < 0.01) in rCa_v_3.2^GAERS^(−25)-expressing cells (393 ± 65 pS/pF, *n* = 21) (Fig. 6F). Remarkably, the electrophysiological data in Fig. 6F appear to be inversely correlated with the biochemical measurements in Fig. 6C, suggesting the possibility that the differences in cell surface expression of the various constructs is mediated by their differential interactions with calnexin. To test this hypothesis, we co-expressed the different rCa_v_3.2 channel variants with the calnexin C-tail peptide to disrupt calnexin regulation of Ca_v_3.2 channels. Preventing calnexin interactions with the channel not only eliminated the differences in current densities observed with the two exon 25 splice isoforms, but importantly also ablated the effects of the GAERS mutation. (Fig. 6G–I). Altogether, these data indicate that calnexin mediated ER retention of Ca_v_3.2 channels is a key determinant of the differential cell surface expression of Ca_v_3.2 III-IV linker splice isoforms and are essential for the functional effects of the GAERS mutation.

## Discussion

Here, we describe a new regulatory pathway for low-voltage-activated T-type Ca^2+^ channels, and provide insights into molecular basis that may underlie the enhancement of T-type Ca^2+^ conductance of thalamic neurons in the Genetic Absence Epilepsy Rat from Strasbourg (GAERS) and other rodent models of absence epilepsy. Our data show that the III-IV linker region of Ca_v_3.2 channels interacts with calnexin to increase ER retention of the channel. Of particular importance, our data revealed that the GAERS mutation critically alters the interaction of calnexin with Ca_v_3.2, resulting in a significant increase in surface expression of the channel, which may account for the elevated T-type Ca^2+^ conductance in GAERS reticular thalamic neurons.

Using a combination of biochemical and electrophysiological approaches, we revealed that binding of calnexin to Ca_v_3.2 has two important consequences. First, it increases the retention of the channel in the ER, resulting in decreased expression of T-type channels at the cell surface without alteration of the total protein levels. Second, it participates in the maturation/folding of Ca_v_3.2 channels as evidenced by an increase of the single channel conductance in the presence of overexpressed calnexin. The increased channel conductance may result from an improved folding of the channel consistent with the chaperone role of calnexin in the maturation of newly synthetized proteins. It is also conceivable that increased ER retention of Ca_v_3.2 alters the glycosylation status of the channel, which in turn may affect ion permeability. Consistent with this idea, we have recently reported that N-linked glycosylation of hCa_v_3.2 at specific loci influences channel permeability^38^. Nonetheless, the net effect of calnexin interactions with the channel is a decrease of Ca_v_3.2 surface expression, consistent with a reduction of the whole cell Ca^2+^ conductance. These results support the existence of a Ca_v_3.2/calnexin signaling complex that is essential for the maturation and sorting of T-type channels to the cell surface, similar to that for Na_v_1.8 channels, CFTR channels, Shaker potassium channels, and other G-protein coupled receptors including acetycholine and dopamine D1 and D2 receptors^39–44^.

Detailed analysis of the molecular determinants of the Ca_v_3.2/calnexin interaction revealed that Ca_v_3.2 interacts with two distinct calnexin regions. In part, Ca_v_3.2 interacts with the ER luminal domain of calnexin. It is well known that this domain is responsible for lectin-like activity of calnexin and glycosylation-dependent interaction with newly synthetized glycoproteins^45^. Although this interaction has not been investigated in more detail in the current study, it likely involves one or more extracellular regions of the channel (i.e. facing the lumen side when the channel is embedded in the membrane of the ER) located in domains I, II and IV that are known to be N-glycosylated^32, 33, 35^. In another part, Ca_v_3.2 interacts with the cytosolic region of calnexin (calnexin C-tail) via its domain III-IV cytosolic linker. We showed that this interaction is sufficient to mediate the retention of Ca_v_3.2 in the ER and to decrease the sorting of channels to the cell surface. Conversely, uncoupling calnexin from its binding site on the channel via coexpression of the calnexin C-tail interfering peptides resulted in a significant increase in the surface expression of the channel. While little is known about the cellular functions of the calnexin C-tail, a role in the control of clathrin-mediated endocytosis of membrane proteins has been proposed^37^. However, our observation that internalization properties of Ca_v_3.2 channels were not affected by the presence of the calnexin C-tail strongly supports the notion that the increased channel surface expression is the direct consequence of a decreased ER retention of the channel upon uncoupling of Ca_v_3.2 from calnexin.

The steady-state expression of T-type channels at the cell surface is governed by a balance between the number of channels arriving at and being removed from the plasma membrane. Hence, our observation that calnexin affects the sorting of T-type channels to the cell surface may have important consequences on neuronal excitability. Indeed, we have revealed a previously unrecognized effect of the GAERS Ca_v_3.2 missense mutation on the trafficking of Ca_v_3.2 channels. Of particular interest is the observation that the GAERS mutation alters the binding of calnexin C-tail to the III-IV linker region of Ca_v_3.2, resulting in a potent increase of the expression of channels at the cell surface. The observation that the GAERS mutation enhances Ca_v_3.2 surface expression by altering calnexin-dependent ER retention of the channel provides a further explanation to the hyperexcitability observed in a subset of GAERS thalamic neurons and believed to underlie absence seizures^25^. A modest increase of Ca_v_3.2 mRNA levels has also been reported in GAERS, but whether this correlates with an increased expression levels of the channel and/or is a result of further underlying genetic alterations remains to be investigated^30^.

It is established that alternative splicing represents an important mechanism that controls the expression and regulation of ion channels including T-type channels^46–51^. Ca_v_3.2 splice variants with distinct biophysical properties have previously been identified in the rat thalamus and that differ by the presence or absence of exon 25 encoding for a small region of the III-IV linker region^6^. Our study revealed that binding of calnexin C-tail on the III-IV linker lacking the exon 25 is significantly decreased, which correlated with an increased expression of the Ca_v_3.2 (−25) channel variant at the cell surface. This observation further supports the notion that Ca_v_3.2/calnexin interaction plays an essential role in the control of surface trafficking of the T-type channel. It also uncovers a new mechanism by which alternative splicing of Ca_v_3.2 can affect channel surface expression. In addition, the surface expression of the Ca_v_3.2 (−25) variant was further augmented in the presence of the GAERS Ca_v_3.2 missense mutation. Of note, the observed splice-variant specific effects of calnexin interactions on Ca_v_3.2 current conductance are quantitatively analogous to that for the previously described splice-variant effects of the GAERS mutation on Ca_v_3.2 recovery from inactivation^6^. Together, these data suggest that GAERS neuronal hyperexcitability and seizure activity may result from a combination of gain-of-functions derived from domain III-IV linker splice variant Ca_v_3.2/calnexin interactions and splice variant specific effects of the GAERS Ca_v_3.2 missense mutation on recovery from inactivation. It remains to be determined whether the GAERS genome possess any further underlying genetic alterations associated with the Ca_v_3.2/calnexin interactions and that could further affect T-type calcium channel functioning or that of other ion channels.

Alteration of T-type channel expression has been linked to various neurological disorders including absence epilepsy. Interestingly, the III-IV linker region of Ca_v_3.2 channels has previously been implicated in the control of channel activation and recovery from inactivation^6, 49, 50^, as well as in the control of channel expression by ubiquitination^52^. Here, we present evidence that the III-IV linker also mediates calnexin-dependent control of channel surface expression. Hence, the III-IV linker region of Ca_v_3.2 channels, albeit relatively short compared to the other intracellular region of the channel, appears to be an essential hub for regulating key aspects of calcium channel trafficking and biophysical properties. The physiological importance of this regulatory hotspot is underscored by its role in chronic pain^52^ and as we show here, seizure disorders.

## Materials and Methods

### Plasmids cDNA constructs

The human wild-type HA-Ca_v_3.2 construct (HA-hCa_v_3.2^WT^) was previously described^53^. This plasmid was used as a template to amplify by PCR the main cytoplasmic regions of the channel (amino- and carboxy-terminal regions, and I-II, II-III, and III-IV linkers), and the PCR products were inserted into the XhoI/HindIII sites of pEGFP-C1 vector. The GFP-tagged III-IV linker construct was used as a template to introduce by site-directed mutagenesis the R1573P and T1606M mutations using overlap extension PCR method, and the PCR products were inserted into the XhoI/HindIII sites of pEGFP-C1 vector. The rat wild-type and GAERS Ca_v_3.2 ± exon 25 variants in pcDNA3.1 were previously described^6^. Sequences encoding the various rat III-IV linkers used in this study were synthetized *in vitro* (GenScript) and subcloned into the XhoI/HindIII sites of pEGFP-C1 vector. To generate mCherry-tagged calnexin constructs, plasmids encoding for the full-length mouse calnexin (CNX^AD^) and calnexin fragments (CNX^AC^ and CNX^BD^) (a generous gift of Dr. Marek Michalak) were used as template for PCR. PCR products encoding for the different domains of calnexin and mCherry were inserted into KpnI/AgeI sites and AgeI/PmeI sites of pcDNA3.1(+) vector, respectively, to generate C-terminal mCherry-tagged calnexin constructs. The PCR primers used to generate those constructed are shown in *Supplementary Information*. All final constructs were verified by sequencing of the full-length cDNAs. The Lck-GFP construct encoding for a membrane-targeted form of GFP was previously described^54^.

### Heterologous expression

Human embryonic kidney tsA-201 cells were grown in Dulbecco’s modified Eagle’s medium (DMEM) containing 10% fetal bovine serum and 1% penicillin/streptomycin (Invitrogen), and maintained under standard conditions at 37 °C in a humidified atmosphere containing 5% CO_2_. Cells were transiently transfected using the calcium phosphate method.

### Patch-clamp electrophysiology

Patch-clamp recordings were performed 72 h after transfection in the whole-cell configuration of the patch-clamp technique at room temperature (22–24 °C) as previously described^55^. Briefly, the bath solution contained (in millimolar): 5 BaCl_2_, 5 KCl, 1 MgCl_2_, 128 NaCl, 10 TEA-Cl, 10 D-glucose, 10 4-(2-hydroxyethyl)-1-piperazineethanesulfonic acid (HEPES) (pH7.2 with NaOH). Patch pipettes had a resistance of 2–4 MΩ when filled with a solution containing (in millimolar): 110 CsCl, 3 Mg-ATP, 0.5 Na-GTP, 2.5 MgCl_2_, 5 D-glucose, 10 EGTA, and 10 HEPES (pH7.4 with CsOH). Whole-cell patch-clamp recordings were performed using an Axopatch 200B amplifier (Axon Instruments). Acquisition and analysis were performed using pClamp 9 and Clampfit 9 software, respectively (Axon Instruments). All traces were corrected online for leak currents, digitized at 10 kHz, and filtered at 2 kHz. The voltage dependence of the peak Ba^2+^ current density was fitted with the following modified Boltzmann equation (1):

1 $$I(V)=Gmax\frac{(V-Vrev)}{1+\exp \frac{(V0.5-V)\,}{k}}$$

with *I*(*V*) being the peak current amplitude at the command potential *V*, *G*_max_ the maximum conductance, *V*_rev_ the reversal potential, *V*_0.5_ the half-activation potential, and *k* the slope factor. The voltage dependence of the whole-cell Ba^2+^ conductance was calculated using the following modified Boltzmann equation (2):

2 $$G(V)=\frac{Gmax}{1+\exp \frac{-(V-V0.5)\,}{k}}$$

with *G*(*V*) being the Ba^2+^ conductance at the command potential *V*.

### Immunoprecipitation

Co-immunoprecipitations were performed 72 h after transfection. Cells were lysed in NP40 lysis buffer (50 mM Tris, 150 mM NaCl, 1% NP40, pH 8.0), incubated for 30 min at 4 °C, and centrifuged for 30 min at 15.000 RPM. The protein concentration was estimated using a Bradford Protein Assay (BioRad). For co-immunoprecipitation experiments, 1 mg/mL total cell lysate was incubated overnight at 4 °C with 2 μg of “catching” antibody (mouse monoclonal anti-GFP (Abcam), rat monoclonal anti-HA (Roche), mouse monoclonal anti-mCherry, or rabbit polyclonal anti-Ca_v_3.2 (Alomone). For co-immunoprecipitation of native proteins, 10% rat brain homogenate was prepared in lysis buffer (50 mM TrisHCl pH 8.0, 300 mM NaCl, 0.5% Triton ×100, 0.5% DOC, Protease Inhibitors without EDTA) and stored as aliquots of 500 ml at −80 °C. For Co-immunoprecipitation, 500 μL of lysis buffer without detergent and 2 μg of “catching” antibody (rabbit polyclonal anti-CNX (Abcam) or rabbit polyclonal anti-Ca_v_3.2 (Santa Cruz)) were added to the 10% brain homogenate samples and incubated overnight at 4 °C. Samples were then incubated with 30 μL (50% slurry) Sepharose G beads for 2 h at 4 °C. Beads were centrifuged for 1 min at low speed, washed three times (20 mM Tris, 300 mM NaCl, 0.1% Tween-20, pH 8.0), and incubated with 30 μL Laemmli buffer.

### SDS-PAGE and immunoblot analysis

Immunoprecipitation samples, or total lysates (25 μg), were separated on 12% SDS-PAGE and transferred onto PVDF membrane (Millipore). For detection of the HA-hCa_v_3.2 channel, the membrane was incubated with a primary rat monoclonal anti-HA antibody (Roche) diluted at 1:1000; GFP-tagged hCa_v_3.2 loops were detected with a primary rat monoclonal anti-GFP antibody (Abcam) diluted at 1:10.000; mCherry-tagged calnexin constructed were detected with a primary mouse monoclonal anti-mCherry antibody (Abcam) diluted at 1:1:1000; native calnexin was detected using a primary rabbit polyclonal anti-calnexin antibody (Abcam) diluted at 1:5000. For detection of native proteins, the following antibodies were used and incubated overnight at 4 °C with the membrane: rabbit polyclonal anti-Ca_v_3.2 antibody (Santa Cruz) diluted at 1:5000; mouse monoclonal anti-BiP antibody diluted at 1:1000 (Santa Cruz); mouse monoclonal anti-Calreticulin antibody (Santa Cruz) diluted at 1:1000; and rabbit polyclonal anti-calnexin antibody (Abcam) diluted at 1:10.000, incubated overnight at 4 °C. Membranes were then washed in PBS/Tween-20 buffer, and incubated with the corresponding secondary HRP-conjugated antibody (Jackson ImmunoResearch) diluted at 1:20.000. For immunoprecipitation controls, membranes were stripped to remove antibodies in a stripping buffer (0.2 M glycine, 1% SDS; pH 2.0) and then reblotted as described above. Immunoreactive bands were detected by enhanced chemiluminescence.

### Surface immunostaining

Twenty-four hours before the experiment, cells expressing HA-Ca_v_3.2 channels were seeded on poly-L-lysine-coated glass coverslips. Cells were incubated for 30 min at 37 °C with a primary monoclonal mouse anti-HA antibody (Abcam) diluted in DMEM at 1:1000, washed with PBS, fixed for 7 min in 4% paraformaldehyde, and blocked for 45 min in blocking buffer (5% FBS in PBS). Cells were then incubated for 1 h at room temperature with a secondary goat polyclonal anti-mouse Alexa488-conjugated antibody (Jackson ImmunoResearch) diluted in blocking buffer at 1:1000, washed, and mounted on microscope glass slides with ProLong Gold mounting medium (Life Technologies). Confocal images were acquired with a Zeiss LSM780 microscope and the field fluorescence intensity was analyzed using ImageJ software.

For internalization studies, cells were incubated with a primary anti-HA antibody as described above, washed, kept at 37 °C for 30, 60, 120, or 180 min to allow internalization of the channel, fixed, and stained with a secondary Alexa488-conjugated antibody to assess the time-dependence of surface expression of the channel. In order to visualize internalized channels, cells were permeabilized with 0.2% Triton X-100 for 10 min before incubation with the secondary antibody.

### Statistical analysis

Data values are presented as mean ± S.E.M. for *n* experiments. Statistical significance was determined using Student’s unpaired *t* test or one-way ANOVA with Tukey’s post tests as indicated in the figure legends: *p < 0.05, **p < 0.01, ***p < 0.001, and NS, statistically not different.

## Electronic supplementary material

Supplementary information
