# Supplementary material for: The Cacna1h mutation in the GAERS model of absence epilepsy enhances T-type Ca2+ currents by altering calnexin-dependent trafficking of Cav3.2 channels
Source: Sci Rep. 2017 Sep 14;7:11513. doi: 10.1038/s41598-017-11591-5 (PMC5599688; doi:10.1038/s41598-017-11591-5)
Supplement: Supplementary file 1 — Supplementary information [file 41598_2017_11591_MOESM1_ESM.pdf]

The *Cacna1h* mutation in the GAERS model of absence epilepsy enhances T-type Ca^{2+} currents by altering calnexin-dependent trafficking of $Ca_v3.2$ channels

Juliane Proft[1], Yuriy Rzhepetskyy[1], Joanna Lazniewska[1], Fang-Xiong Zhang[2], Stuart M. Cain[3], Terrance P. Snutch[3], Gerald W. Zamponi[2,*], Norbert Weiss[1,*]

Supplementary Information

Table S1. PCR primers used to generate the cDNA constructs used in this study.

Figure S1. Calreticulin and BiP do not associate with $Ca_v3.2$ channels.
Figure S2. The human calnexin modulates all T-type channel isoforms.
Figure S3. Calnexin affects $Ca_v3.2$ single channel properties
Figure S4. Calnexin does not affect surface expression of Lck-GFP.
Figure S5. Calnexin ER transmembrane domain is not required for calnexin Ctail to associate with $hCa_v3.2$ III-IV linker.

Supplementary Methods

For nonstationary noise analysis, experimental points were generated with a set of 100 individual current traces elicited by a 150 ms long depolarizing step to -20 mV from a holding potential of -100 mV. The variance-mean analysis was performed using ANA software developed by Dr. Michael Pusch at the Institute of Biophysics in Genova, Italy (http://users.ge.ibf.cnr.it/pusch/programs-mik.htm). The fit of the variance-mean histogram is performed with the following equation (1):

$$(1)\ \sigma^2 = \sigma_0^2 + i*(1+o\hat{}2)*(I\text{-}leak) - (I\text{-}leak)^2/N$$

where σ_0^2 is the background variance, *leak* is the leak current, *i* is the single channel current, *N* the number of functional channels, *o* is the "fraction open channel noise" (normally = 0), and *I* the independent variable, the mean current.

Supplementary Table

Table S1. PCR primers used to generate the cDNA constructs used in this study.

**Constructs**		**Forward primer**	**Reverse primer**
**pEGFP-C1-hCa$_v$3.2 loops**	Nter	ATTCTCGAGCCATGACCGAGGGCGCACGG	GAGAAGCTTTCATGGGTTGCAGACCAGCC
	I-II	ATTCTCGAGTAACGCAGTTCTCGGAGACG	GAGAAGCTTTCATGCTGTCCACGATGCGGC
	II-III	ATTCTCGAGTAGAGGGCTTCCAGGCGGAG	GAGAAGCTTTCACCTTCTGGCAGGAGACGC
	III-IV	ATTCTCGAGTAAACTTCCACAAGTGCCG	GAGAAGCTTTCAGCTGGTGCACAGCGAGTG
	Cter	ATTCTCGAGTAGAGGAGAGCAACAAGGAG	GAGAAGCTTTCACACGGGGTCATCTGCAC
**pEGFP-C1-hCa$_v$3.2 III-IV**	R1573P	CGGCGGCCCGAGGAGAAGCGGCTGCGGCGCCTAGAGAGGAGG	CAGCCGCTTCTCCTCGGGCCGCCGCGCCTCCTCCGCCTCCTGGTG
	T1606M	GCCGACTACTCGCCCATGCGCCGCTCCATTCACTCGCAGC	GCGGCGCATGGGCGAGTAGTCGGCATAGTAGGGCCGGCGCTG
**pcDNA3.1-CNX-mCherry**		ggggtaccATGGAAGGGAAGTGGTTACT	ccaccggtCTCTCTTCGTGGCTTTCTGT

Supplementary Figures

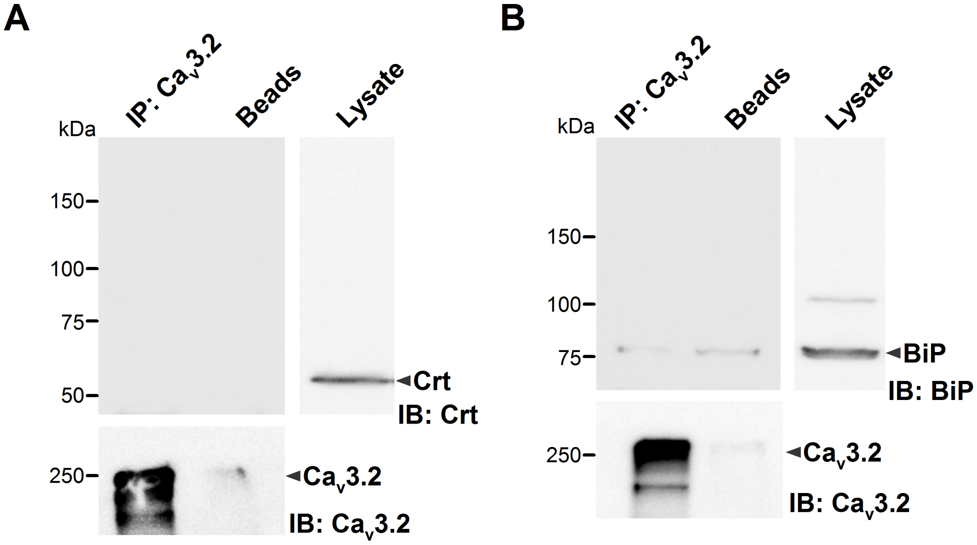

Figure S1. Calreticulin and BiP do not associate with $Ca_v3.2$ channels. (**A**) Co-immunoprecipitation of calreticulin (Crt) from rat brain homogenate with specific $Ca_v3.2$ antibody. (**B**) Co-immunoprecipitation of BiP from rat brain homogenate with specific $Ca_v3.2$ antibody.

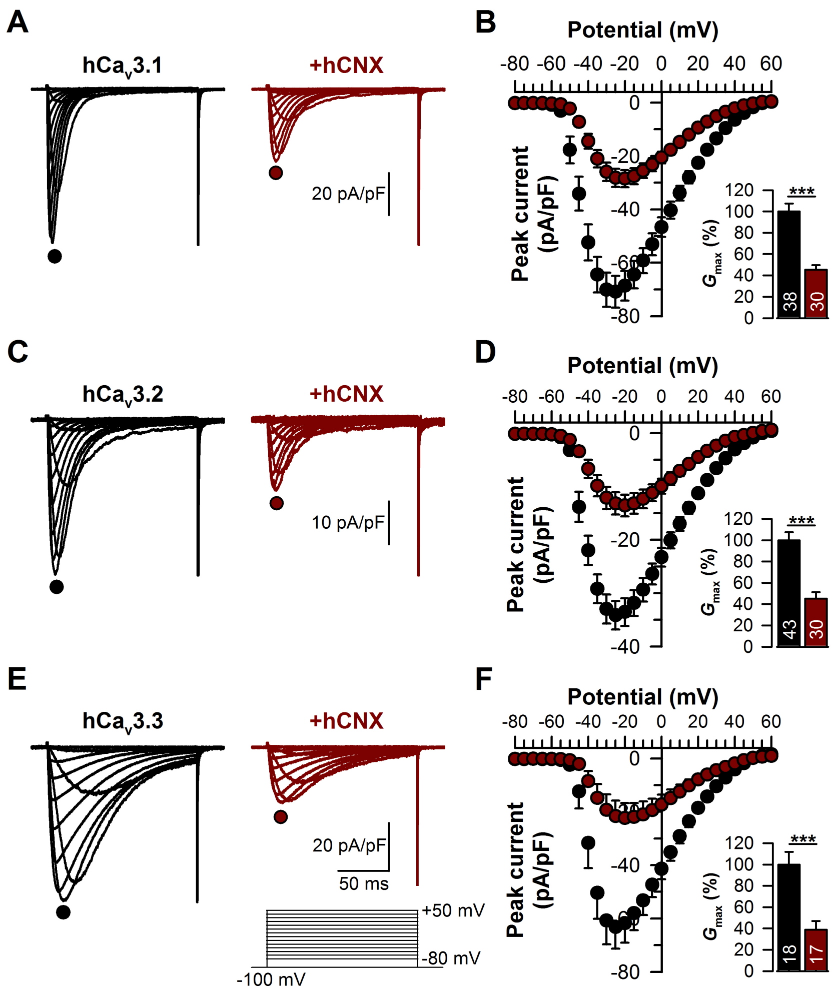

Figure S2. The human calnexin modulates all T-type channel isoforms. Representative Ba^{2+} current traces recorded from tsA-201 cells expressing human $Ca_V3.1$ (**A**), $Ca_V3.2$ (**C**) and $Ca_V3.3$ channels (**E**) alone (left panels) and co-transfected with the human CNX (right panels) in response to 150-ms depolarizing steps varied from -80 mV to +50 mV from a holding potential of -100 mV. Corresponding mean current/voltage relationships for $hCa_V3.1$ (**B**), $hCa_V3.2$ (**D**) and $hCa_V3.3$ channels (**F**) expressed alone (black circles) and in the presence of hCNX (red circles). The *insets* indicate the corresponding maximal conductance G_{max} expressed in percentage of $hCa_V3.x$-expressing cells. Data were analyzed by Student's unpaired *t* test; *** $p<0.001$.

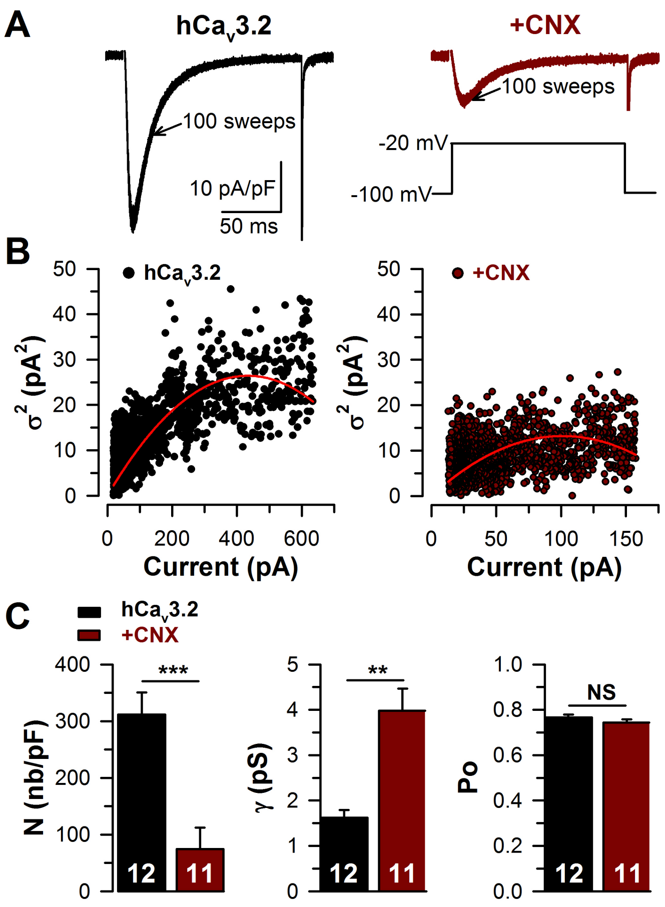

Figure S3. Calnexin affects hCa$_v$3.2 single channel properties. (**A**) Representative sets of 100 current traces recorded from hCa$_v$3.2-HA- (left panel) and hCa$_v$3.2-HA/CNX-expressing tsA-201 cells (right panel) elicited by successive 150-ms depolarizing steps to -20 mV from a holding potential of -100 mV and used to perform non-stationary noise analysis of hCa$_v$3.2 channels. (**B**) Corresponding current variance plotted against mean current for data shown in (A), fitted with the function $\sigma^2 = \sigma_0^2 + i*(1+o\text{^}2)*(I\text{-}leak) - (I\text{-}leak)^2/N$ where σ_0^2 is the background variance, *leak* is the leak current, *i* is the single channel current, *N* the number of functional channels, *o* is the "fraction open channel noise" (normally = 0), and *I* the independent variable, the mean current. (**C**) Summary of noise analysis parameters including the channel density (*N*), the single channel conductance (*g*), and the open probability (*Po*) for hCa$_v$3.2-HA- (black bars, *n*=12) and hCa$_v$3.2-HA/CNX-expressing cells (dark red bars, *n*=11). Data are presented as mean ± SEM and were analyzed by Student's unpaired *t* test; NS not significant, ** $p<0.005$, *** $p<0.001$.

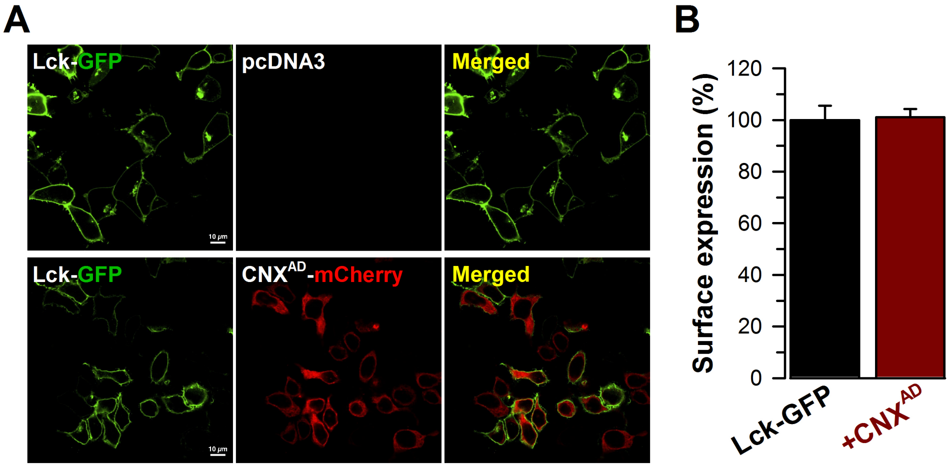

Figure S4. Calnexin does not affect surface expression of Lck-GFP. (**A**) Low magnification confocal images of live tsA-201 cells expressing the plasma membrane LcK-GFP fusion protein (green, left panels) in the absence (middle top panel) and presence of CNX-mCherry (red, middle bottom panel). Overlaid images are also shown (left panels). (**B**) Corresponding mean surface expression of Lck-GFP in the absence (black bar, n=3) and presence of CNX-mCherry (red bar, n=3).

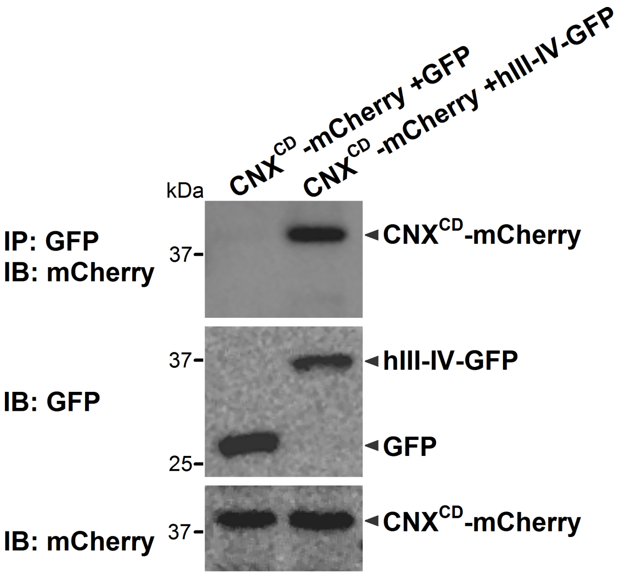

Figure S5. Calnexin ER transmembrane domain is not required for calnexin Ctail to associate with $hCa_v3.2$ III-IV linker. Co-immunoprecipitation of CNX C-tail (CNX^{CD}-mCherry) from tsA-201 cells with $hCa_v3.2$ III-IV linker. The upper panel shows the result of the co-immunoprecipitation of CNX^{CD}-mCherry with $hCa_v3.2$ III-IV linker using an anti-GFP antibody. Middle panel show the immunoblots of $hCa_v3.2$ III-IV linker using anti-GFP antibody. The lower panel shows the Immunoblot of CNX^{CD}-mCherry using an anti-mCherry antibody.
